# MicroRNA-29b-3p enhances radiosensitivity through modulating WISP1-mediated mitochondrial apoptosis in prostate cancer cells

**DOI:** 10.7150/jca.48216

**Published:** 2020-09-12

**Authors:** Aihong Mao, Jinzhou Tang, Deping Tang, Fang Wang, Shiqi Liao, Hongxia Yuan, Caiping Tian, Chao Sun, Jing Si, Hong Zhang, Xiaojun Xia

**Affiliations:** 1 Gansu Provincial Academic Institute for Medical Research, Lanzhou, China.; 2 Institute of Modern Physics, Chinese Academy of Sciences, Lanzhou, China.; 3 School of Chemical & Biological Engineering, Lanzhou Jiaotong University, Lanzhou 730070, PR China.; 4 Gansu Provincial Cancer Hospital, Lanzhou, China.

**Keywords:** MiR-29b-3p, WISP1, radiosensitivity, apoptosis, prostate cancer

## Abstract

Radiotherapy is frequently applied for clinically localized prostate cancer while its efficacy could be significantly hindered by radioresistance. MicroRNAs (miRNAs) are important regulators in mediating cellular responses to ionizing radiation (IR), and strongly associate with radiosensitivity in many cancers. In this study, enhancement of radiosensitivity by miR-29b-3p was demonstrated in prostate cancer cell line LNCaP *in vitro*. Results showed that miR-29b-3p expression was significantly upregulated in response to IR from both X-rays and carbon ion irradiations. Knockdown of miR-29b-3p resulted in radioresistance while overexpression of miR-29b-3p led to increased radiosensitivity (showing reduced cell viability, suppressed cell proliferation and decreased colony formation). In addition, miR-29b-3p was found to directly target Wnt1-inducible-signaling protein 1 (WISP1). Inhibition of WISP1 facilitated the mitochondrial apoptosis pathway through suppressing Bcl-XL expression while activating caspase-3 and poly (ADP-ribose) polymerase (PARP). The results indicated that miR-29b-3p was a radiosensitizing miRNAs and could enhance radiosensitivity of LNCaP cells by targeting WISP1. These findings suggested a novel treatment to overcome radioresistance in prostate cancer patients, especially those with higher levels of the WISP1 expression.

## Introduction

Prostate cancer is a prevalent cancer among men. In the United States, the number of new cases was ~191,930 whereas deaths account for 33,330 in 2020, which is showing an increasing incidence over the past number of years 1. Radiotherapy is a frequently applied therapeutic intervention for localized prostate cancer. However, most patients typically develop radioresistance following a period of radiotherapy, leading to reduced efficacy 2. Therefore, identification of novel approaches or agents to reverse the radioresistance of prostate cancer cells and improve the efficacy of radiotherapy is urgently required.

WISP1 (Wnt-1 inducible signaling pathway protein-1) belongs to the CCN family, which is regulated by the Wnt-1/β-catenin pathway 3. Overexpression of WISP1 not only could potentiate transformation and tumorigenesis, but also closely associated with poor prognosis in colon cancer, glioblastoma and oral squamous cell carcinoma 4-6. A number of studies have demonstrated that WISP1 could facilitate prostate cancer cell growth and bone metastasis 7-9. In addition, studies on esophageal carcinoma suggested that WISP1 was involved in mediating radioresistance 10-12, whilst the Wnt-1/β-catenin pathway, which lies upstream of WISP1, was demonstrated to mediate radioresistance in glioblastoma 13 and breast cancer 14. Therefore, targeting WISP1 may be a potentially useful approach for overcoming the radioresistance of prostate cancer cells.

Over the last decade, accumulating evidence has showed that microRNAs (miRNAs) serve pivotal roles in tumorigenesis and progression 15. The dysregulation of miRNAs has been detected in many cancers 16. Recently, we found several miRNAs, such as miR-449a and miR-16-5p, dysregulated in prostate cancer tissue or cells, where they served critical roles in response to ionizing radiation (IR) and associated strongly with radiosensitivity in prostate cancer cells 17-19. These findings suggested that miRNAs have potential as novel targets for prostate cancer radiotherapy.

MicroRNA-29b (miR-29b) is a member of the miR-29 family (miR-29a, b, and c), which locates on two distinct chromosomes 1q32 and 7q32 20. Aberrant expression profiles of miR-29b has been found in many cancers, where the role of miR-29b in the development and progression of many types of malignancies has been extensively studied 21. In prostate cancer, reduced expression of miR-29b or deletion was observed in tumor tissues or/and cell lines 22,23. The expression of miR-29b was found to associate strongly with etiology, classification, progression, and prognosis of patients with prostate cancer 23. Emerging evidences demonstrate that miR-29b inhibits prostate cancer cell proliferation and invasion by regulating different targets, including MCL-1, MMP-2 24,25, DNMT3B and AKT3 26. MiR-29b was identified as a regulator of epithelial-mesenchymal transition (EMT), which was involved in prostate cancers metastasis 27 and chemoresistance 26,28. Although the function of miR-29b as a tumor suppressor was reported on multiple occasions to critically influence cancer progression and therapeutic outcome, it is still unclear whether WISP1 and miR-29b interact in response to IR. And whether miR-29b regulates radiosensitivity by targeting WISP1 in prostate cancer cells also remains to be fully elucidated.

Therefore, in this study, we investigated the expression and function of miR-29b-3p in response to IR in prostate cancer cells. Our results demonstrated that miR-29b-3p enhances IR-induced cell apoptosis and sensitizes LNCaP cells to X-rays irradiation by targeting WISP1. The results highlighted a previously unrecognized mechanism involving miR-29b-3p and WISP1 in response to IR, and suggested a novel treatment option for prostate cancer patients, especially for those with higher levels of the WISP1 expression.

## Materials and Methods

### Cell culture and irradiation treatment

Human prostate cancer cell line LNCaP was obtained from the Cell Bank of Type Culture Collection of the Chinese Academy of Sciences. Cells were cultured in RPMI-1640 medium with 10% FBS (Hyclone; GE Healthcare Life Sciences) and kept in a humidified 5% CO_2_ incubator at 37 °C.

X-rays were generated by an X-ray machine (Faxitron RX-650) with 100 kVp, at a dose rate of 0.835 Gy/min. Carbon ion irradiation was performed on the Heavy Ion Research Facility in Lanzhou (HIRFL; Institute of Modern Physics, Chinese Academy of Sciences, Lanzhou, China) with energy 80.55 MeV/u and linear energy transfer was 59.75 keV/µm, and dose rate was 2 Gy/min.

### RNA isolation and miRNA array analysis

LNCaP cells with 80% confluence were seeded into 60-mm dishes and stabilized overnight at 37 °C and 5% CO_2_. The cells were exposed to X-rays or carbon ion irradiation at a dose of 0.5 or 4 Gy at room temperature. 24 h after irradiation, total RNA samples were isolated using TRIzol® reagent (Invitrogen, Thermo Fisher Scientific, Inc.) and sent to Shanghai GeneChem Co., Ltd. for miRNA OneArray analysis, as previously described 19.

### Reverse transcription-quantitative PCR (RT-qPCR)

Total RNA was isolated using Trizol reagent and reverse transcription reaction was performed using Mir-X™ miRNA First Strand Synthesis Kit (Takara Bio., Inc.) according to manufacturer's protocol. Real-Time PCR was carried out to measure the expression of miR-29b-3p using Mir-X™ miRNA qRT-PCR Kit (Takara Bio., Inc.) with a specific sense primer (Table S1). WISP1 mRNA expression analysis was performed using Prime Script™ RT reagent Kit and gDNA Eraser and SYBR Advantage qPCR Premix (Takara Bio., Inc.) with specific primers (Table S2). The amplification reactions were performed using the QuantStudio™ 5 Real-Time PCR System (Thermo Fisher Scientific, Inc.). The relative expression levels of miRNA and mRNA were evaluated using the 2^-ΔΔCt^ method. RNU6 and GAPDH were used as references, respectively.

### miRNA mimics and inhibitors transfection

LNCaP cells with 30-40% confluence were incubated with antibiotic free RPMI-1640 medium for 24 h at 37 °C. Transfection was performed using the Trans IT®-2020 transfection reagent (Mirus Bio LLC) according to manufacturer's protocol. The sequences of the miR-29b-3p mimics (miR-29b-3p), inhibitors (anti-miR-29b-3p) and corresponding negative control (miR-con or anti-con) (RIBO Bio., Guangzhou) were presented in Table S3. MiR-29b-3p mimics and inhibitors were transfected in LNCaP cells at a final concentration of 50 and 100 nM, respectively.

### Cell viability assay

LNCaP cells were seeded into 96-well plates at 1×10^3^ cells per well and incubated overnight at 37 °C. Cells were transfected with either miR-29b-3p mimics or inhibitors. After 24 h, cells were irradiated with X-rays at dose of 0, 1, 2, 4, 6 or 8 Gy at room temperature. 24 h after IR, cells were incubated with 5 μg/μL MTT (Sigma-Aldrich) at 37 °C for 4 h, following which the supernatant was removed and 150 μL DMSO (Sigma-Aldrich) was added into each well to dissolve the formazan crystals. The absorbance value was recorded at 490 nm using a microplate reader (Tecan Infinite M200; Tecan Group, Ltd.).

In addition*,* cell viability of LNCaP following miRNA mimics or inhibitor transfection and 4Gy X-rays irradiation was also measured using the Cell Counting Kit-8 (CCK-8; Dojindo Molecular Technologies, Inc.) at 24, 48 and 72 h after IR at 37 °C, as described previously 18.

### Clone survival assay

As described above, the irradiated cells were seeded in 60 mm dishes with 2×10^3^ cells. Cells were incubated for 13 days and colonies were fixed using 100% methanol and stained with 0.5% crystal violet (Sigma-Aldrich) for 30 min at room temperature. Only colonies consisting of ≥50 cells were counted for each treatment group.

### Luciferase assay

To construct WISP1 3′-untranslated region (UTR)-luciferase plasmid, the 425bp WISP1 3′-UTR fragments containing the miR-29b-binding (WISP1-3′-UTR-WT) or WISP1 3′-UTR-mutated (WISP1-3′-UTR-Mut) site of miR-29b (Table S4) were inserted into the multiple cloning site of pGL3-basic vector, which contains a luciferase reporter gene. DNA sequencing assay identified the sequence (BGI-Tech., Wuhan). 293T cells were seeded into 48-well plates with 50% confluence 200 ng/μL constructed luciferase vectors and 50 nM miR-29b-3p mimics or negative control (miR-con) were cotransfected into 293T cells using Lipofectamine 2000 Transfection reagent (Invitrogen, Carlsbad, CA). The cells were lysed 48 h after transfection. Firefly and Renilla luciferase activities were measured using the Dual-Luciferase® Reporter Assay Kit (Promega Coporation) on the microplate reader according to manufacturer's protocol (Tecan Infinite M200; Tecan Group, Ltd.). Renilla luciferase was used to normalize the firefly luciferase enzyme activity.

### Apoptosis assays

LNCaP cells with 30% confluence were transfected with either miR-29b-3p mimics or inhibitors for 24 h and exposed to 4 Gy X-ray. 1×10^6^ cells were harvested 24 h after IR and stained with FITC-Annexin V and propidium iodide (PI) at final cell density of 1×10^3^ cells/μL to measure the number of apoptotic/necrotic cells using Annexin V/PI staining kit according to manufacturer's protocol (BD Biosciences). Apoptosis cells were measured by BD FACSVerse™ and analyzed using the BD FlowJo® software (BD Biosciences).

### Western blot

The cells were lysed using RIPA buffer supplemented with 1% PMSF (Beyotime Bio.) on ice. Bicinchoninic acid protein assay was performed to determine protein concentration (Pierce; Thermo Fisher Scientific, Inc). 20 μg total protein was fractionated and loaded onto 10% SDS-PAGE and transferred to 0.45 μm PVDF membrane. The membranes were blocked with 5% not-fat milk in buffer for 2 h at room temperature, and followed by incubation with primary antibodies: WISP1 (ab155654; 1:1,000; Abcam), Bcl-XL (ab32370; 1:5,000; Abcam), cleaved-caspase 3 (9661; 1:1,000; Cell Signaling Technology, Inc.), caspase 3 (9662; 1:1,000; Cell Signaling Technology, Inc.), PARP (9532; 1:1,000; Cell Signaling Technology, Inc.), cleaved PARP (5625; 1:1,000; Cell Signaling Technology, Inc.) and β-actin (AP0060; 1:5,000; Bioworld Tech., Inc.) overnight at 4 °C. Horseradish peroxidase-conjugated anti-rabbit IgG secondary antibody was incubated with membranes for 2 h at room temperature. The protein blots were visualized using a chemiluminescence kit (EMD Millipore) and photographed using AI680 (Alpha Innotech Corporation). The relative expression of proteins was quantified using Image Quant TL software (version 8.1; GE Healthcare Life Sciences).

### Statistical analysis

Statistical analysis was performed using two-tailed Student's *t*-test and two-way ANOVA. Comparing statistical significance of >2 groups was analyzed using Tukey's test. Data are presented as means ± SD (n=3). *P*<0.05 was considered significant difference.

## Results

### miR-29b-3p expression is increased in response to IR in LNCaP cells

**Figure 1:**
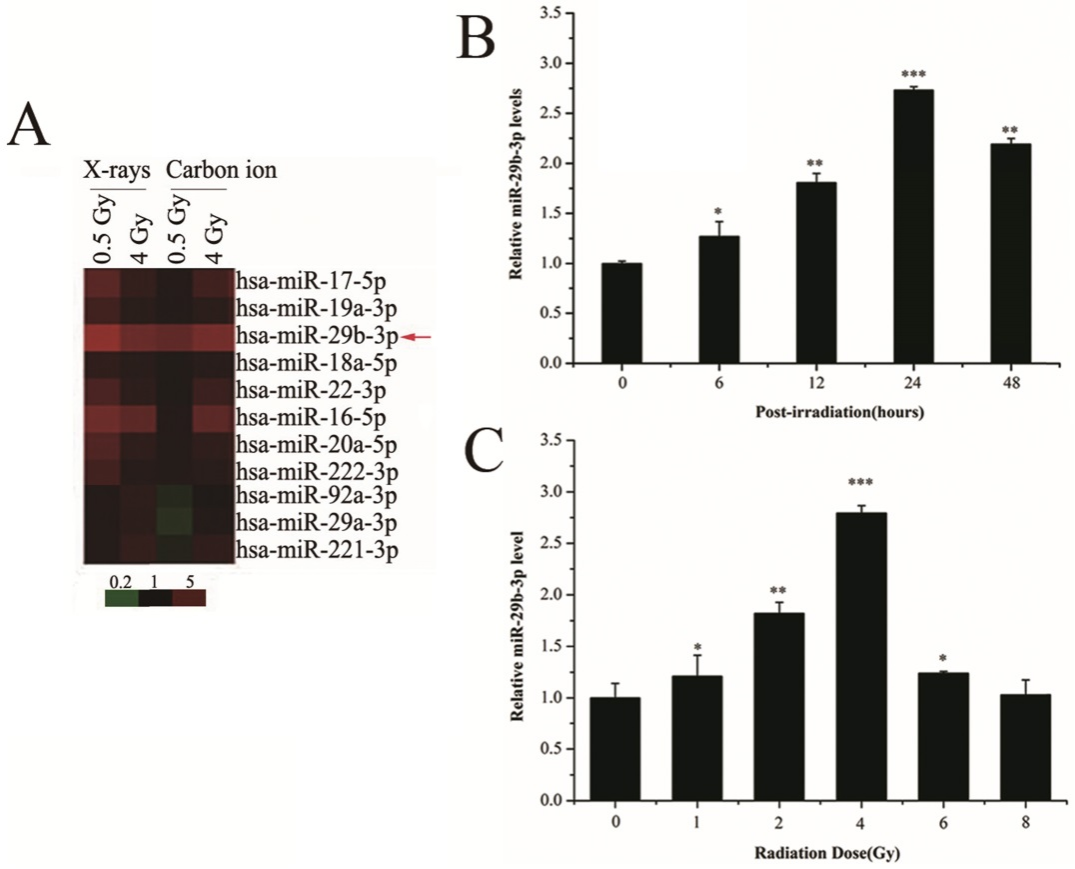
miR-29b-3p expression is increased in response to IR in LNCaP cells. (**A**) A mini heat map of the miRNA expression profile in LNCaP cells exposed to either 0.5 or 4 Gy X-rays or carbon ion beam irradiation. Red arrow indicates the location of miR-29b-3p, the expression of which is upregulated in response to IR. (**B**) The levels of miR-29b-3p expression in LNCaP cells at indicated time points following exposure to 4 Gy X-ray irradiation. (**C**) Levels of miR-29b-3p expression in LNCaP cells 24 h after irradiation with indicated doses of X-rays. *P<0.05, **P<0.01 and ***P<0.001 vs. 0 h or 0 Gy.

To measure the expression alteration of miRNAs in response to IR, LNCaP cells were exposed to 0.5 or 4 Gy X-rays or carbon ions. The expression of miR-29b-3p was upregulated in LNCaP cells following IR (Fig. 1A). To verify this finding, RT-qPCR was performed and showed that miR-29b-3p expression reached its maximum at 24 h with 4 Gy X-ray irradiation (Fig. 1B and C). These results indicated that miR-29b-3p was upregulated and could be involved in response to IR in LNCaP cells.

### miR-29b-3p knockdown attenuates the response of LNCaP cells to IR

**Figure 2:**
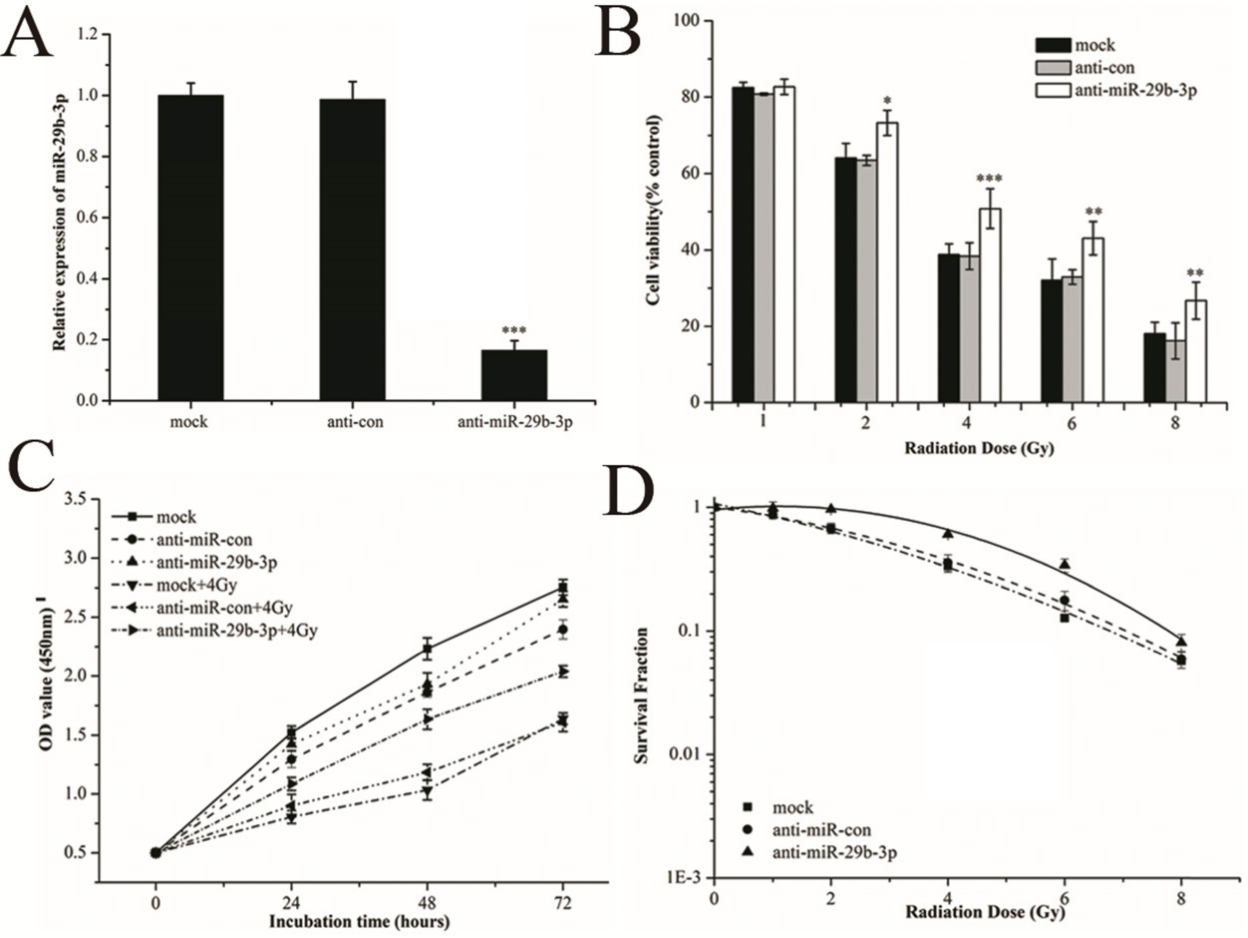
miR-29b-3p knockdown attenuates response of LNCaP cells to IR. (**A**) The relative levels of miR-29b-3p expression 48 h after transfection with miR-29b-3p inhibitors or negative control. (**B**) Cell viability, (**C**) Cell proliferation and (**D**) clonogenic ability in LNCaP cells were measured following transfection with anti-miR-29b-3p and X-ray exposure. *P<0.05, **P<0.01 and ***P<0.001 vs. negative control group (anti-con).

To investigate the radiobiological role of miR-29b-3p in LNCaP cells, miR-29b-3p was knocked down by transfecting anti-miR-29b-3p into LNCaP cells. 48h after transfection, the expression of miR-29b-3p was significantly reduced (Fig. 2A). After knockdown of miR-29b-3p and X-rays irradiation, cell viability was significantly higher than that in the negative control group (anti-con) (Fig. 2B), whilst IR-induced growth inhibition was attenuated (Fig. 2C). Clone survival of LNCaP cells was increased (Fig. 2D and Figure S1). The results suggested that knockdown of miR-29b-3p reduced the cellular response to IR, leading to radioresistance.

### Overexpression of miR-29b-3p enhances the radiosensitivity of LNCaP cells

**Figure 3:**
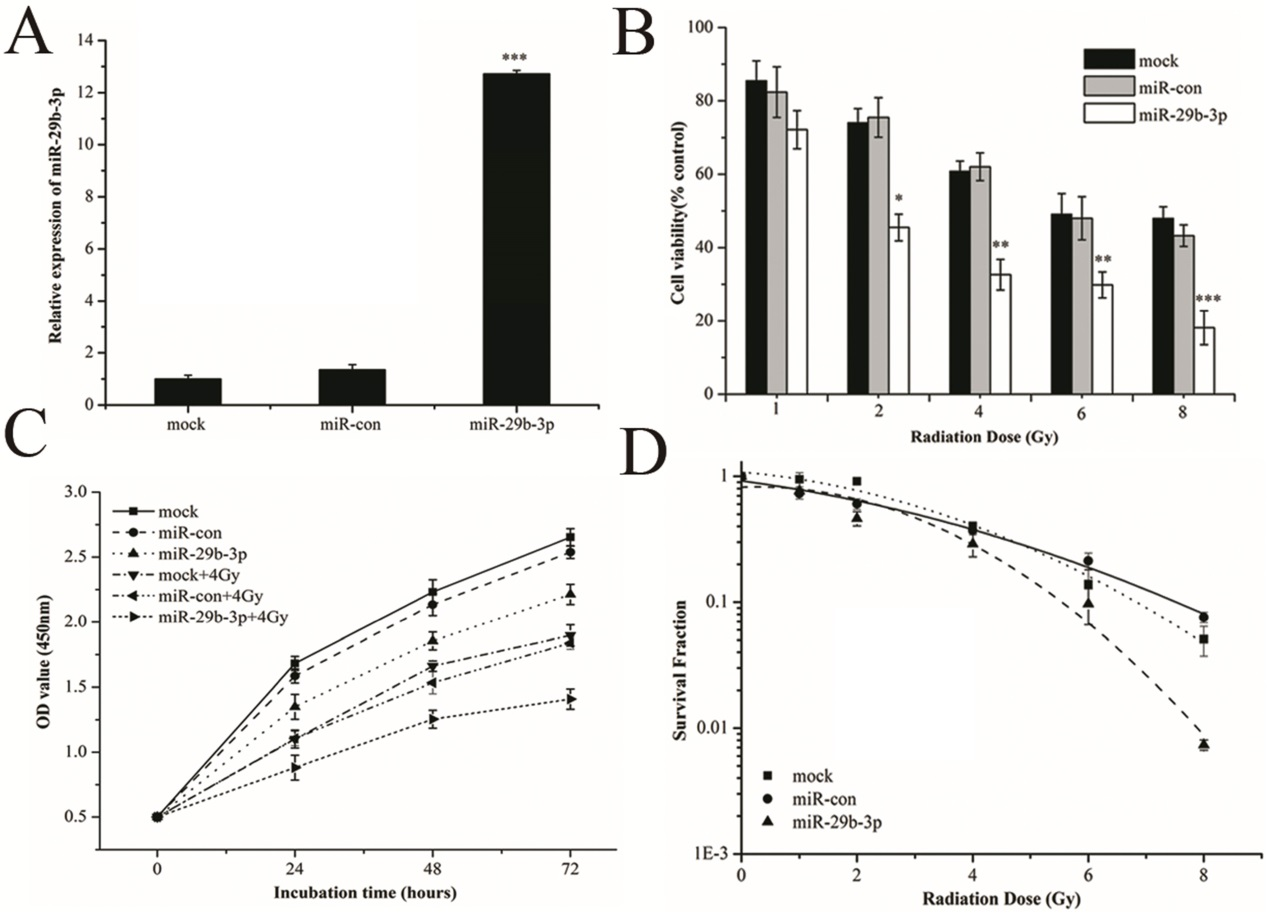
Overexpression of miR-29b-3p enhances radiosensitivity in LNCaP cells. (**A**) Relative levels of miR-29b-3p expression 48 h following transfection with the miR-29b-3p mimic or negative control. (**B**) Cell viability, (**C**) Cell proliferation and (**D**) Clonogenic survival was measured in LNCaP cells overexpressing miR-29b-3p and treatment with indicated doses of X-ray irradiation. *P<0.05, **P<0.01 and ***P<0.001 vs. negative control group (miR-con).

To verify the role of miR-29b-3p in response to IR, miR-29b-3p mimics were transfected into LNCaP cells to overexpress it. As shown in Fig.3A, miR-29b-3p mimics markedly increased the expression of miR-29b-3p. Overexpression of miR-29b-3p reduced cell viability (Fig. 3B), suppressed proliferation (Fig. 3C) and reduced clone survival (Fig. 3D and Figure S2) in LNCaP cells after exposure to X-rays. These findings demonstrated that overexpression of miR-29b-3p enhanced radiosensitivity of LNCaP cells.

### miR-29b-3p suppresses WISP1 expression by directly targeting the 3'-UTR of WISP1 mRNA

**Figure 4:**
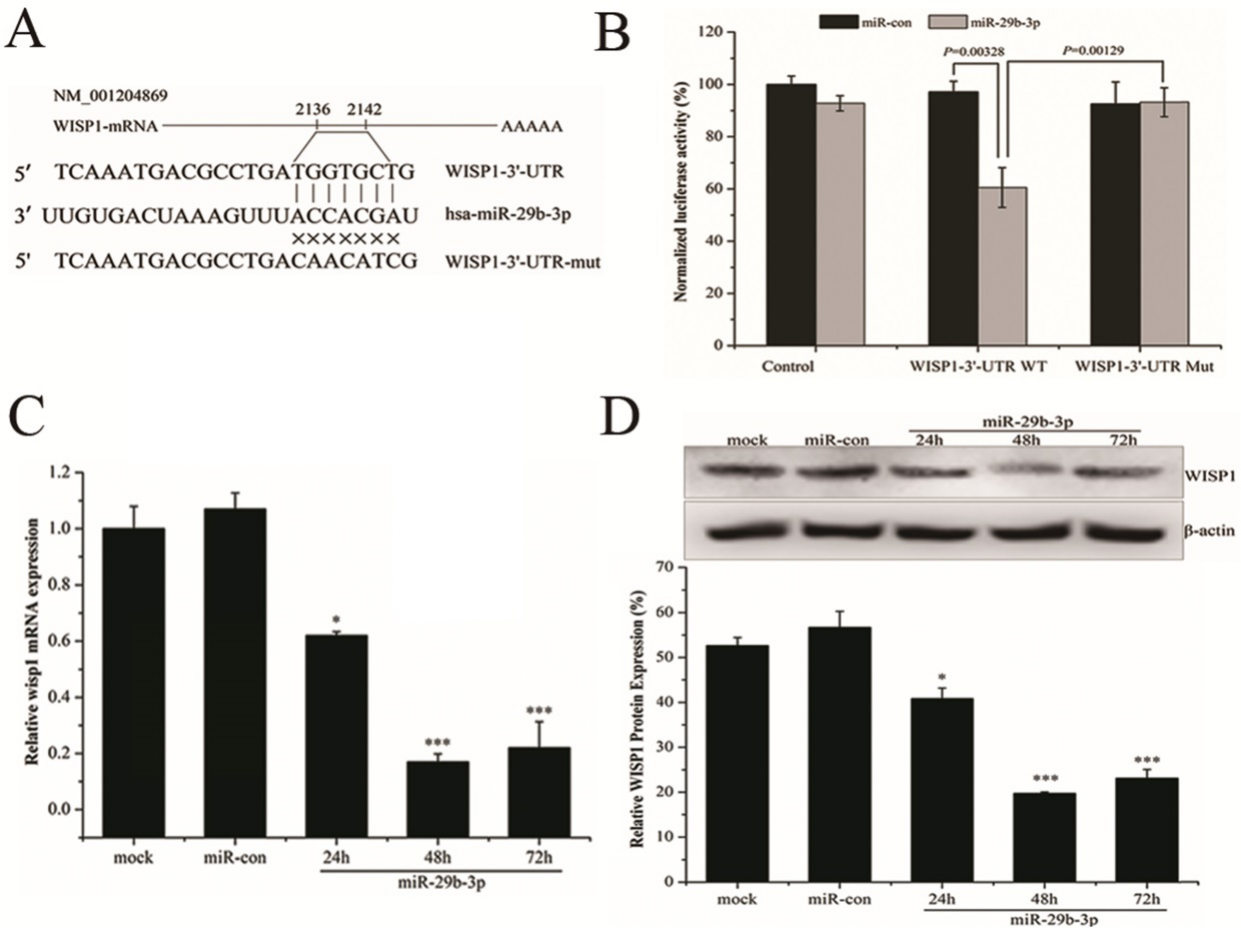
miR-29b-3p suppresses WISP1 expression by targeting 3'-UTR of WISP1. (**A**) Putative miR-29b-3p binding site in the 3'-UTR of human WISP1 mRNA. Sequence of the mature miR-29b-3p was aligned against the corresponding target site on the WT and the MUT 3'-UTR of the WISP1 mRNA. (**B**) Luciferase activity was measured 48 h after cotransfection with the pGL3 vector encoding WT or MUT WISP1 3'-UTR and miR-con or miR-29b-3p mimics. Relative expression of WISP1 (**C**) mRNA and (**D**) protein in LNCaP cells following transfection with miR-29b-3p mimics or miR-con. *P<0.05 and ***P<0.001 vs. negative control group (miR-con).

miRNAs function to regulate gene expression through binding to mRNA targets. In terms of miR-29b-3p, target prediction using three databases (TargetScan, miRanda, RNA22) revealed a perfect match between the seed region of miR-29b-3p and the 3′-UTR of WISP1 mRNA (Fig.4A). To conform the prediction, pGL3-WISP1-3′-UTR-WT and pGL3-WISP1-3′-UTR-Mut vectors were constructed. As shown in Fig. 4B, miR-29b-3p mimics significantly reduced the luciferase activity in 293T cells cotransfected with pGL3-WISP1-3′-UTR-WT, but did not affect that in cells cotransfected with the pGL3-WISP1-3′-UTR-Mut vector. The result supported the prediction that miR-29b-3p targeted WISP1 through directly binding to its 3′-UTR. To further verify the direct relationship between WISP1 and miR-29b-3p, WISP1 mRNA and protein expression were measured after transfecting of miR-29b-3p mimics. Compared with miR-con group, the expression levels of both WISP1 mRNA and protein were significantly reduced in cells transfected with the miR-29b-3p mimics (Fig. 4C and D), validating miR-29b-3p targeting downregulated WISP1 expression.

### miR-29b-3p overexpression enhances IR-induced apoptosis by modulating the WISP1-mediated mitochondrial apoptosis pathway

**Figure 5:**
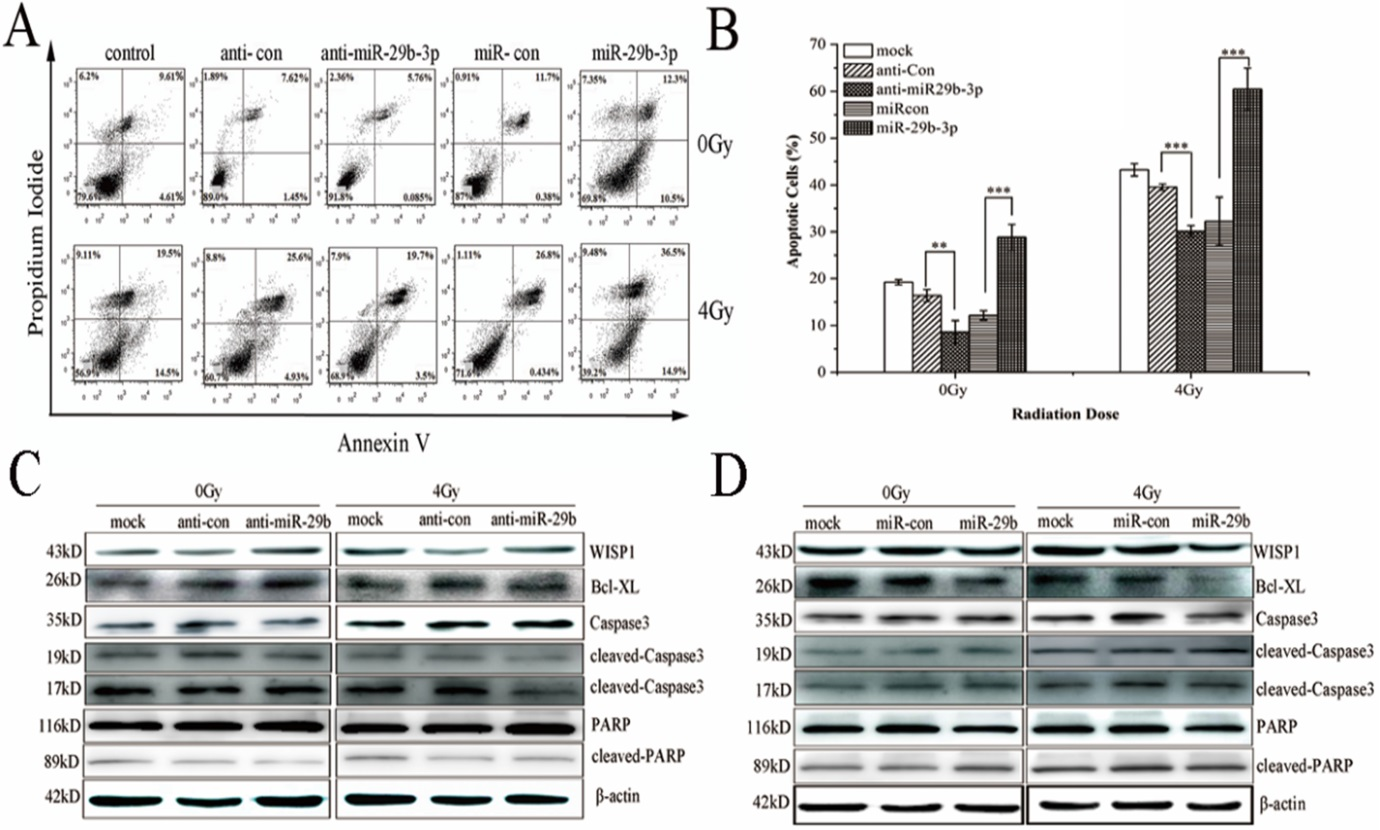
miR-29b-3p enhances IR-induced apoptosis by suppressing WISP1 expression and activating the mitochondrial apoptosis pathway. (**A**) Representative flow cytometry dot plots showing apoptotic LNCaP cells transfeced with miR-29b-3p inhibitors or mimics 24h before 4 Gy X-rays irradiation. (**B**) Quantification of (A). (**C**) miR-29b-3p knockdown increased the expression levels of WISP1 and Bcl-XL whilst blocking the activation of caspase 3 and PARP in response to IR. (**D**) miR-29b-3p overexpression reduced WISP1 and Bcl-XL expression, whilst inducing the activation of caspase3 and PARP in response to IR. **P<0.01 and ***P<0.001 vs. negative control group.

Overexpression of WISP1 could accelerate cell growth and inhibit apoptosis by suppressing the activation of caspase 3 and release of cytochrome C 29. Therefore, the effect of miR-29b-3p on IR-induced apoptosis may be associated with WISP1-mediated mitochondrial apoptosis pathway. Indeed, as shown in Fig. 5A and B, overexpression of miR-29b-3p dramatically increased IR-induced apoptosis in LNCaP cells. Interestingly, miR-29b-3p itself could also increase apoptosis in the absence of IR when compared with cells transfected with miR-con. While knockdown of miR-29b-3p had the opposite effect, that is to say, anti-miR-29b-3p attenuated IR-caused apoptosis.

To unravel the possible molecular mechanism underlying the miR-29b-3p-mediated enhancement in IR-induced apoptosis, expression of a number of protein components related with mitochondrial apoptosis pathway was measured. Overexpression of miR-29b-3p markedly reduced expression of WISP1 and Bcl-XL, but increased the activation of caspase 3 and PARP. X-ray irradiation further reduced expression of WISP1 and Bcl-XL, whereas activated caspase3 and PARP (Fig. 5D). By contrast, transfecting miR-29b-3p inhibitors into LNCaP cells resulted in the opposite effects (Fig. 5C). The results suggested that miR-29b-3p enhanced IR-induced apoptosis by suppressing WISP1 expression, activating mitochondrial apoptosis pathway through modulating the Bcl-XL expression and activation of caspase 3 and PARP.

## Discussion

Accumulating evidence has demonstrated that the expression level of miR-29b is lower in variety of malignancies and cancer cell lines than that in normal tissues or cell lines 21. miR-29b expression has been correlated with clinicopathological characteristics in patients with colorectal cancer 30-32, breast cancer 33, and osteosarcoma 34 patients, suggesting its potential to serve as a biomarker for diagnosis and prognosis of cancer. miR-29b has been documented to negatively affect tumorigenesis and cancer progression by regulating cell proliferation, survival, and metastasis 28, Whilst overexpression of miR-29b can also inhibit tumorigenesis 35 and angiogenesis 36-38. Moreover, miR-29b can reverse drug resistance in ovarian cancer 39,40 and colorectal cancer 41, as well as radioresistance in cervical cancer 42. In this study, we found that overexpression of miR-29b-3p reduced cell viability, suppressed cell proliferation, and decreased colony formation, resulting in increased sensitivity of LNCaP cells to X-rays irradiation. Based on the experimental and clinical evidence, miR-29b can be potentially explored as a targeted therapeutic agent.

Prostate cancer is the most common cancer among man, where radiotherapy is currently the only definitive treatment option for patients with localized prostate cancer 2. However, resistance to radiotherapy remains a major obstacle that limits the efficacy of this treatment. To improve the outcome of radiotherapy and enhance radiosensitivity of prostate cancer cells, understanding the mechanisms underlying cellular radioresistance is currently a hot topic of research. In the present study, miR-29b-3p expression was found to be significantly increased in response to X-rays and carbon ions irradiation in LNCaP cells, suggesting that miR-29b-3p participate in the response to IR. Gain- and loss-of-function experiments next verified that overexpression of miR-29b-3p reduced cell viability, suppressed cell proliferation, and decreased colony formation, resulting in increased sensitivity of LNCaP cells to X-rays irradiation. By contrast, knockdown of miR-29b-3p expression resulted in the opposite effects. To the best of our knowledge, this is the first work showing miR-29b-3p upregulated in response to IR and overexpression of miR-29b-3p enhanced the radiosensitivity of LNCaP cells. Unfortunately, in this study, the function of miR-29b-3p in response to only X-ray irradiation was investigated due to restrictions on the use of the carbon ion beam radiation source at the HIRFL. The mechanistic function of miR-29b-3p in mediating the response to carbon ion radiation requires further study.

WISP-1, as a member of the CCN family of growth factors, plays a critical role in tumorigenesis and development of various cancers 3,43. WISP1 has been demonstrated to involve in cell proliferation, apoptosis, invasion and metastasis 44. Supporting this, a number of studies revealed that WISP1 was associated with the formation and evolution of lung cancer 45, hepatocellular carcinoma 46 and colorectal cancer 4. The ectopic expression of WISP1 has also been observed in a number of cancers, including prostate cancer 7. Inhibition of WISP1 function using neutralizing antibodies reduced prostate cancer cell growth and inhibited metastasis to bone 7. Consistent with these findings, osteoblast-derived WISP1 has also been shown to promote migration and enhance cell adhesion to the bone through VCAM-1/α4β1 integrin pathway in prostate cancer cells 8,9. Therefore, WISP1 can serve as a potential therapeutic target to prostate cancer patients. Interestingly, WISP1 was found to contribute fractionated IR-induced radioresistance in xenograft tumor models, where targeting WISP1 using antibody or siRNA sensitized esophageal carcinoma cells to IR 10-12. In addition, IR-induced aberrant activation of the Wnt/β-catenin pathway was associated with radioresistance 13,14. In our study, WISP1 was predicted and subsequently verified to be a direct target of miR-29b-3p. MiR-29b-3p enhanced sensitivity of LNCaP cells to X-rays irradiation by directly inhibiting WISP1 expression.

Previous study has suggested WISP1 attenuated p53-mediated apoptosis by upregulating the expression of anti-apoptotic Bcl-XL but blocking activation of caspase 3 29. This study showed that inhibition of WISP1 by miR-29b-3p overexpression markedly reduced Bcl-XL expression but significantly activated caspase 3 and PARP in LNCaP cells following 4 Gy X-rays irradiation. It was speculated that miR-29b-3p enhanced IR-induced apoptosis by suppressing WISP1 expression, leading to activation of the mitochondrial apoptosis pathway.

## Conclusion

**Figure 6:**
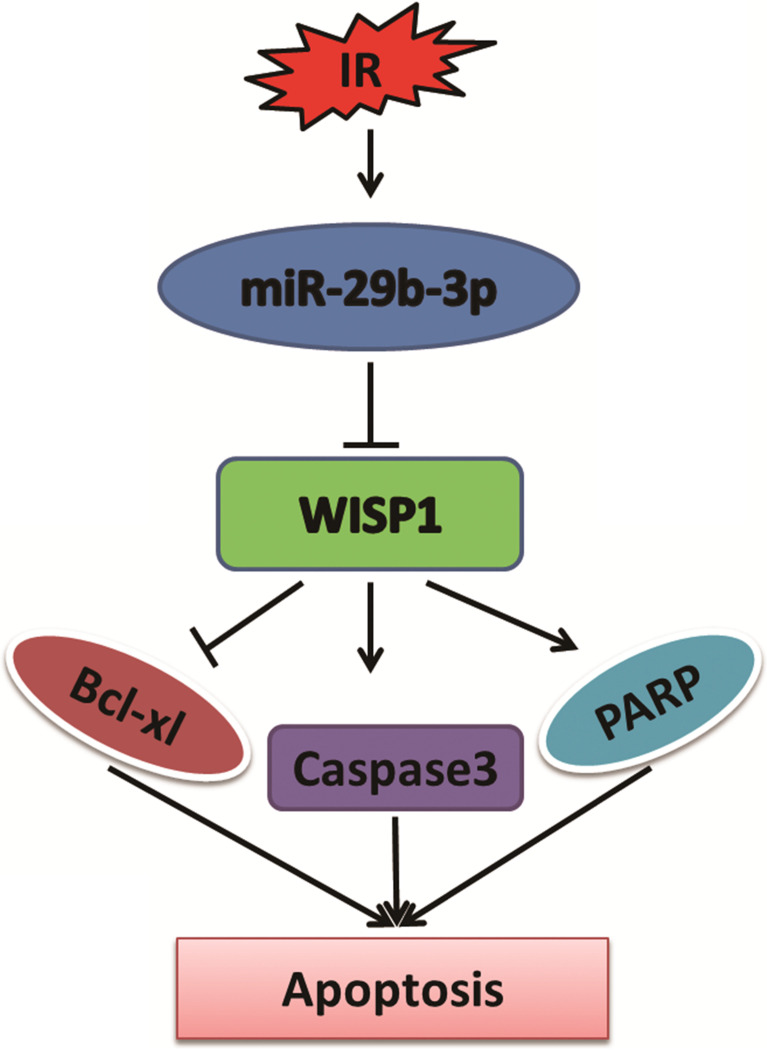
The function of miR-29b-3p and WISP1 in IR-induced apoptosis. miR-29b-3p expression is upregulated in LNCaP cells in response to IR. Elevated miR-29b-3p suppresses WISP1 expression by directly targeting the 3'-UTR of WISP1 mRNA, which then activates the mitochondrial apoptosis pathway by suppressing Bcl-XL expression, but activating Caspase 3 and PARP, resulting in increased LNCaP cell apoptosis.

In summary, miR-29b-3p was significantly upregulated in response to IR. Overexpression of miR-29b-3p enhanced sensitivity of LNCaP cells to X-rays irradiation. WISP1 was identified as a direct target of miR-29b-3p. miR-29b-3p enhanced the radiosensitivity through modulating WISP1-mediated mitochondrial apoptosis (showing reduced Bcl-XL and activation of caspase 3 and PARP) (Fig. 6). The results revealed a novel mechanism of miR-29b-3p enhanced radiosensitivity by target-regulating WISP1 expression in response to IR. These findings may provide an alternative treatment to overcome radioresistance for prostate cancer patients, in particular for those with higher level of WISP1 expression.

## Supplementary Material

Supplementary figures and tables.
