# Supplementary material for: MicroRNA-29b-3p enhances radiosensitivity through modulating WISP1-mediated mitochondrial apoptosis in prostate cancer cells
Source: J Cancer. 2020 Sep 12;11(21):6356–64. doi: 10.7150/jca.48216 (PMC7532503; doi:10.7150/jca.48216)
Supplement: Supplementary file 1 — Supplementary figures and tables. [file jcav11p6356s1.pdf]

Supplementary Tables and Figures

Table S1 Primer sequences of miR-29b-3p and RNU6

Gene		Sequence
miR-29b-3p		5'-TAGCACCATTTGAAATCAGTGTT-3'
RNU6	F<br>R	5'-CTCGCTTCGGCAGCACA-3'<br>5'-AACGCTTCACGAATTTG-3'

Table S2 Primer sequences for WISP1 and GADPH

Gene		Sequence
Wisp1	F<br>R	5'-CGAGGTACGCAATAGGAGTGTG-3'<br>5'-CACGTGCAGTTGTACTTGCAGTTAG-3'
GADPH	F<br>R	5'-TCGCTCTCTGCTCCTCCTGTTC-3'<br>5'-CGCCCAATACGACCAAATCC-3'

Table S3 miRNA/Oligonucleotide sequences for expression miR-29b

Gene	Sequence
miR-29b-3p mimics	5'-UAGCACCAUUUGAAAUCAGUGUU-3'<br>3'-AUCGUGGUAAACUUUAGUCACAA-5'
miR-con	5'-UUUGUACUACACAAAAGUACUG-3'<br>3'-AAACAUGAUGUGUUUUCAUGAC-5'
anti-miR-29b-3p	5'-UAGCACCAUUUGAAAUCAGUGUU-3'
anti-miR-con	5'- AAACAUGAUGUGUUUUCAUGAC -3'

Table S4 Oligonucleotide sequences for WISP1-3'UTR and WISP1-3'UTR mutant

Oligonucleotide	Sequence
WISP1-3'UTR	5'-TCTAGAATTCCCTCTTTCCCATCGGAACCAGCTCTCATCACACATTTAAAAG<br>ATGATTCTGTTTACCCAATGCTGCATATTGAATGTTGTGTAGTTATTCACAGGGA<br>ATTCTGTGCAGTGTGCAGAGAGATTCCTAAACGGGAAAAGGACTGGGAATAC<br>ATCCTCCTTACTGTGACCTCCCCAAAACCTAGTCCAGTGCAAGGTATACAGTGG<br>TGCTCATTAAATACTTGATGAATACAGGAAGCTGTGCATGTGTTCCTACTTTTAT<br>TCGAAGCTCTCTTCTTCCAAAGCTACATGAAAATAGAATTTTAACAGTCAAAAT<br>TTTATATTAAGTGCCTTAGCAAAAGAGACATTTAATATTTCAAA GAAATGCATA<br>TGTATGTATA CATATATTTG TGTATGCGTA TGCAAGAATTTCTAGA-3'
WISP1-3'UTR MUT	5'-TCTAGAATTCCCTCTTTCCCATCGGAACCAGCTCTCATCACACATTTAAAAGA<br>TGATTCTGTTTACCCAATGCTGCATATTGAATGTTGTGTAGTTATTCACAGGGAA<br>TTCTGTGCAGTGTGCAGAGAGATTCCTAAACGGGAAAAGGACTGGGAATACA<br>TCCTCCTTACTGTGACCTCCCCAAAACCTAGTCCAGTGCAAGGTATACAGCAAC<br>ATCCATTAAATACTTGATGAATACAGGAAGCTGTGCATGTGTTCCTACTTTTATT<br>CGAAGCTCTCTTCTTCCAAAGCTACATGAAAATAGAATTTTAACAGTCAAAATT<br>TTATATTAAGTGCCTTAGCAAAAGAGACATTTAATATTTCAAAGAAATGCATATG<br>TATGTATACATATATTTGTGTATGCGTATGCAAGAATTTCTAGA-3'

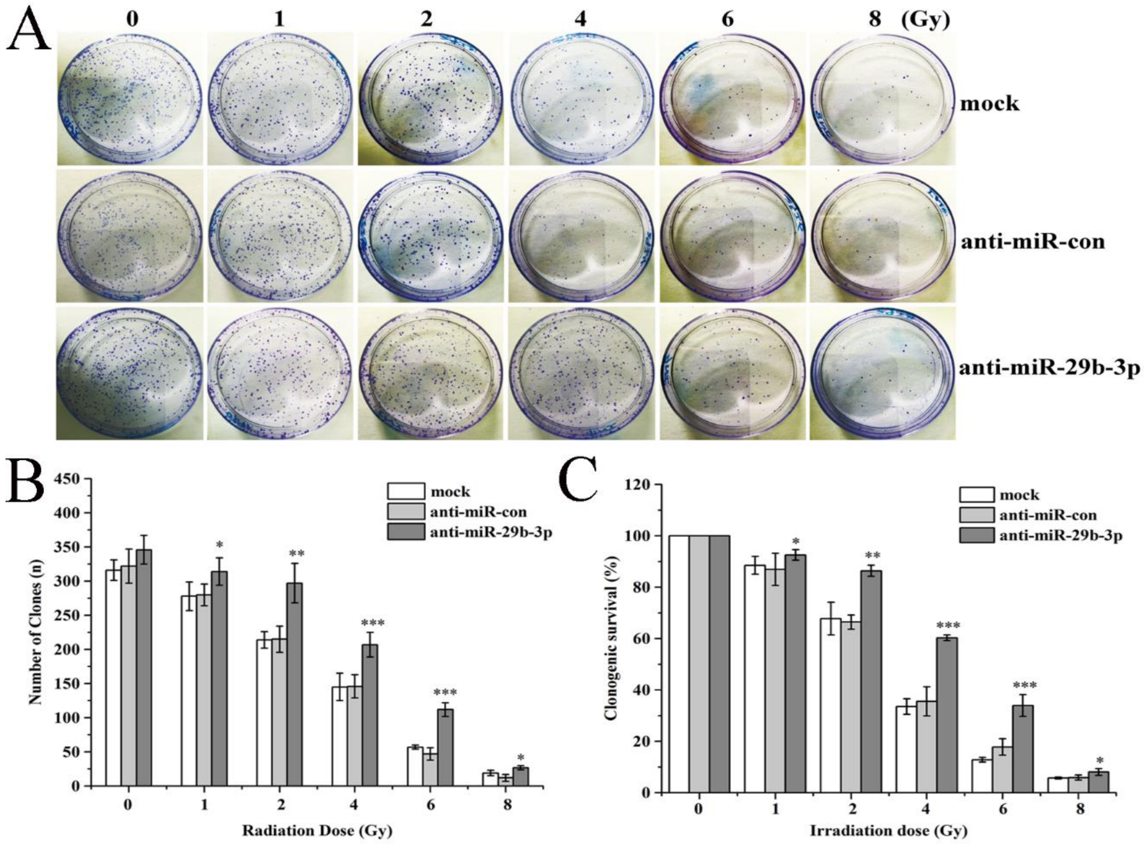

Figure S1. Knockdown of miR-29b-3p attenuates IR-induced inhibition of clone survival in LNCaP cell. (A) Photographs of clone formed by LNCaP following X-rays irradiation. (B) The number of clones. (C) Relative percent of clonogenic survival. $^{*}P<0.05$, $^{**}P<0.01$ and $^{***}P<0.001$ vs. negative control group (anti-con).

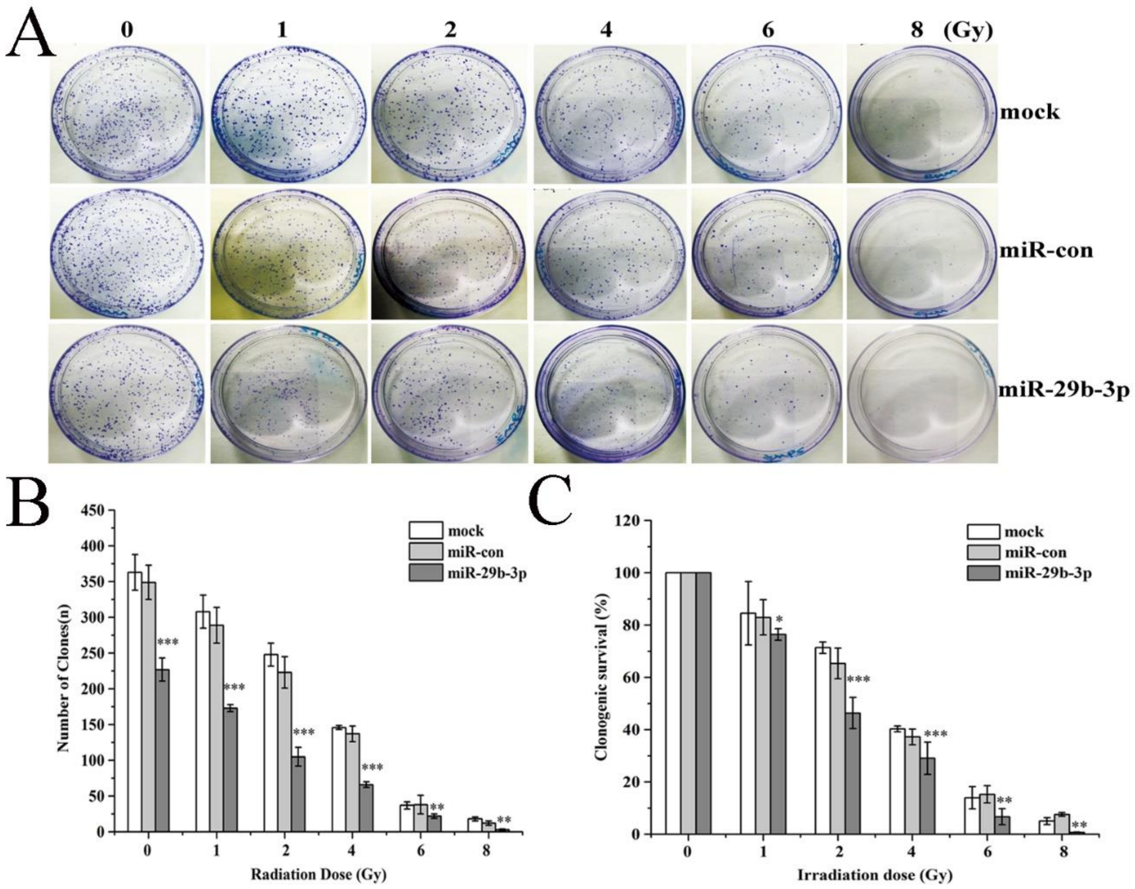

Figure S2. Overexpression of miR-29b-3p enhances IR-induced inhibition of clone survival in LNCaP cell. (A) Photographs of clone formed by LNCaP following X-rays irradiation. (B) The number of clones. (C) Relative percent of clonogenic survival. $^{*}P<0.05$, $^{**}P<0.01$ and $^{***}P<0.001$ vs. negative control group (miR-con).
